# Child and adolescent health – Resources and potential of the international ‘Health Behaviour in School-aged Children (HBSC)’ study

**DOI:** 10.25646/11866

**Published:** 2024-03-04

**Authors:** Irene Moor, Martin Weber, Matthias Richter, for the HBSC Study Group Germany

**Affiliations:** 1 Martin Luther University Halle-Wittenberg, Medical Faculty, Interdisciplinary Centre for Health Sciences (PZG), Institute of Medical Sociology Halle (Saale), Germany; 2 WHO Office on Quality of Care and Patient Safety, World Health Organization Regional Office for Europe (WHO/Europe) Athens, Greece; 3 Technical University of Munich, School of Medicine and Health, Department of Health and Sport Sciences, Chair of Social Determinants of Health Munich, Germany

The initiation of the Health Behaviour in School-aged Children (HBSC) study in 1982 by researchers from Finland, Norway, and England in cooperation with the World Health Organization (WHO) Europe laid the foundation for one of the largest studies on child and adolescent health worldwide. When the first survey was undertaken in 1983/84 in only five countries [1, 2], the researchers at that time would certainly not have thought that this study would be so successful for over 40 years, or that, nowadays 51 countries in Europe and North America and over 450 scientists would participate. The idea at the time was as simple as ingenious: they asked themselves how the health of children and adolescents in their respective country is, in comparison to other countries. How are adolescents doing in comparison to those in other countries? Do other countries have similar problems? The need for a cross-national study emerged from these questions, for which the study would provide reliable data. A study that uses the same methodological approach as well as the same instruments across all countries. In addition, the HBSC Study Group wanted to understand different core areas of child and adolescent health. Therefore, it had to be a broad study that would examine how health and health behaviour of children and adolescents develop over time by regular surveys and at the same time take into account contextual factors that are important for young people’s health, such as family or school. Since the foundation of the study, the international HBSC network has published over 1,300 publications and cooperated at international level with key stakeholders in child and adolescent health, such as the WHO and UNICEF, resulting in additional reports on current topics such as obesity, substance use, and children’s health during the COVID-19 pandemic (see www.hbsc.org under Publications and Reports).

The HBSC study is one of the main sources of data for WHO/Europe. The problems identified are transformed into ‘action points’ in the European strategy for child and adolescent health and wellbeing, which provides the WHO member states (53 in Europe and Central Asia) with recommendations for action [3, 4]. The cross-national results are of high value: why are young people in other countries doing better, where have health outcomes improved or deteriorated? This is the particular benefit of HBSC compared to purely national studies.

Thanks to Professor Dr. Klaus Hurrelmann, Germany has been participating in this study since the 1990s. Until then, health reporting on child and adolescent health was fragmented. At that time, representative information on a national level was largely lacking, and it was often limited to specific regions or topics. One notable exception were the Drug Affinity Studies conducted by the Federal Centre for Health Education (BZgA), which were able to provide representative information on substance use [5]. Although the HBSC study Germany was initially limited to North Rhine-Westphalia, several federal states were added to the study over time, resulting in a nationwide study since 2009/10.

In addition, the Robert Koch Institute launched the German Health Interview and Examination Survey for Children and Adolescents (KiGGS) in the early 2000s, in which more than 10,000 children and adolescents aged 0 to 17 repeatedly took part throughout Germany. In contrast to HBSC, the KiGGS study does not take place in a school setting and also collects objective test and examination data, in addition to questionnaire data, e.g. motor skills tests, measuring and weighing, blood samples. These two study approaches complement each other with regard to child and adolescent health.

Over the course of 30 years, the HBSC study Germany has not only grown into a nationwide study, but also into a veritable treasure trove of data. With each survey cycle, survey data from around 5,000 students aged around 11, 13, and 15 years can be analysed. Information from over 20,000 students is thus available for the present trend analysis (2009/10 – 2022), as described in the methodology article by Winter & Moor et al. in this issue of the Journal of Health Monitoring.

The HBSC study is therefore an extremely important source of data on the health of students in Germany and internationally. In this issue, we have focussed on the results from the current 2022 survey as well as trends over time in Germany, in order to outline health developments from 2009/10 to 2022 and thus contribute to health monitoring. The HBSC Study Group Germany currently consists of seven locations that have jointly conducted the nationwide HBSC study without external financial support. With their respective expertise, this issue was able to cover a broad spectrum of topics relating to child and adolescent health: from subjective health and psychosomatic complaints (Reiß & Behn et al.) to health literacy (Sendatzki & Helmchen et al.), bullying (Fischer et al.), and physical activity (Bucksch et al.), to trends in health inequalities (Moor et al.). The findings show whether efforts in health promotion and prevention have been successful in recent years and what current challenges lie ahead.

The foundations for health in adulthood lie in childhood and adolescence. In addition, the COVID-19 pandemic has shown the impact that crises can have on the young generation. The lessons learned in recent years show that we should regularly examine what adolescents need to grow up healthy and which challenges require special consideration. While substance use was one of the main causes for concern in the 1990s and 2000s, today’s challenges in child and adolescent health include impaired mental health, dealing with crises, the influence of social media, climate change, and increasing social and health inequalities. Regular monitoring of child and adolescent health using complementary studies is therefore essential. The HBSC study sees it as their responsibility to contribute the necessary information in order to give children and adolescents a voice.
